# Let’s Address Hatred by Identifying Its Various Aspects and Appreciating the Usefulness of Different Tools and Interventions Aimed at Tackling Its Numerous Forms and Manifestations: A Systematic Review

**DOI:** 10.1192/bjo.2025.10223

**Published:** 2025-06-20

**Authors:** Salman Shafiq

**Affiliations:** Lancashire and South Cumbria NHS Foundation Trust, Preston, United Kingdom

## Abstract

**Aims:** Hatred can manifest in several ways. This article aims to explore what information can be obtained from the medical literature to address a variety of manifestations and expressions of hatred.

**Methods:** A systematic search of the medical literature from the PubMed medical library was used to identify articles dealing with hatred. A review of 1226 articles from 2015 to the date of data collection was performed. 87 of these discussed the issue of addressing hatred by various means. Full text search of these 87 articles was carried out. Data collected was interpreted utilising thematic analysis.

**Results:** The thematic analysis of data suggests that there are three major ways of addressing hatred: a need to understand various aspects related to hatred; the usefulness of and/or unhelpfulness of various tools, methods or means to address hatred; and lastly, utilising various interventions to address hatred.

**Conclusion:** As there are several forms and manifestations of hatred, with unique background and presentation, there cannot be a single method or suggestion that can be provided to address hatred. Medical researchers concur that hatred exists, and several related aspects require attention. Useful details of several tools, methods, means and strategies are offered that may help to tackle numerous forms and manifestations of hatred. Furthermore, they share with us evidence in favour of several interventions that they found helpful in addressing hatred.

